# Supplementary material for: Complete Mapping of Interacting Charging States in Single Coupled Colloidal Quantum Dot Molecules
Source: ACS Nano. 2022 Mar 15;16(4):5566–76. doi: 10.1021/acsnano.1c10329 (PMC9047002; doi:10.1021/acsnano.1c10329)
Supplement: Supplementary file 1 — nn1c10329_si_001.pdf [file nn1c10329_si_001.pdf]

Supplementary Information for

Complete mapping of interacting charging states in single coupled colloidal quantum dot molecules

Yossef E. Panfil, Jiabin Cui, Somnath Koley & Uri Banin

Institute of Chemistry and the Center for Nanoscience and Nanotechnology, The Hebrew University of Jerusalem, Jerusalem 91904, Israel.

Additional examples of simultaneous spectral shifts of the two QD comprising the CQDM.

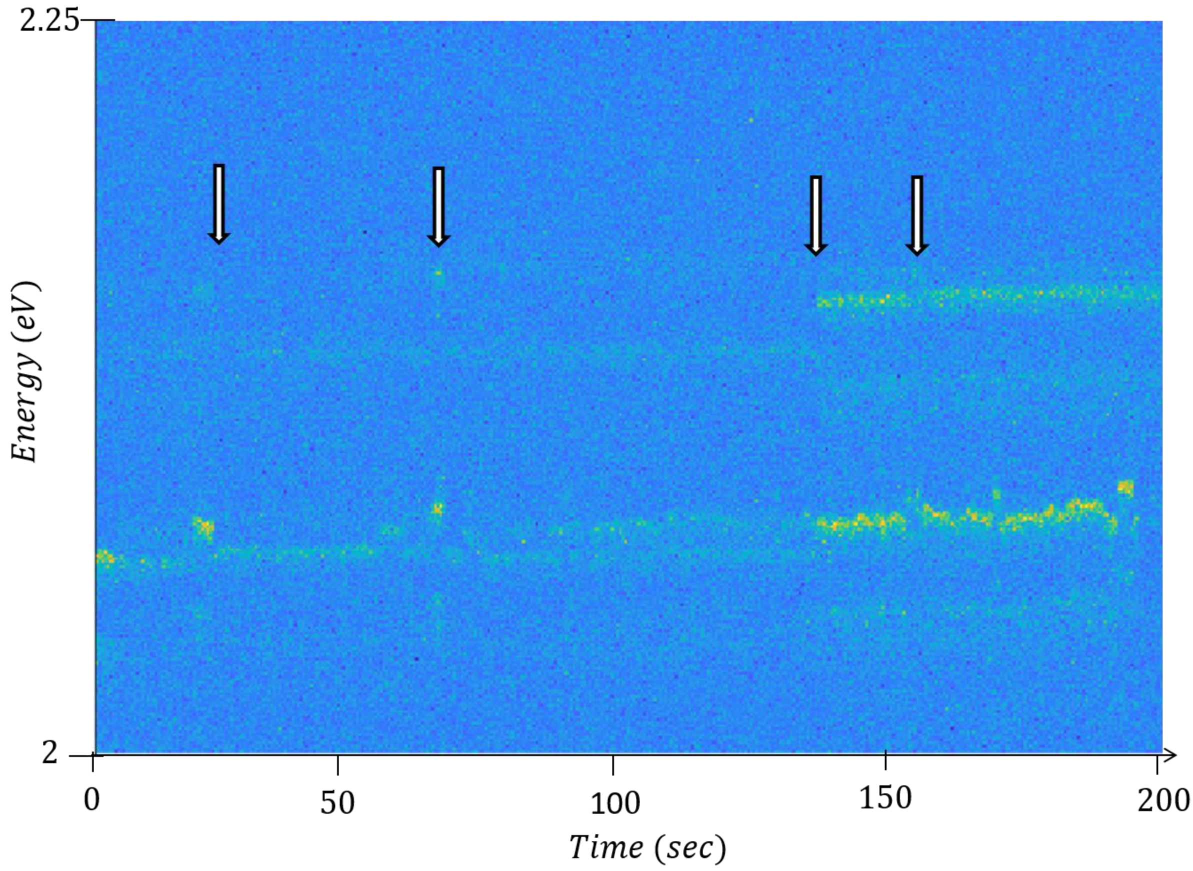

Figure S1. Simultaneous spectral shifts of the two QD comprising the CQDM. Example 1. The arrows marking the times when simultaneous spectral shifts are seen.

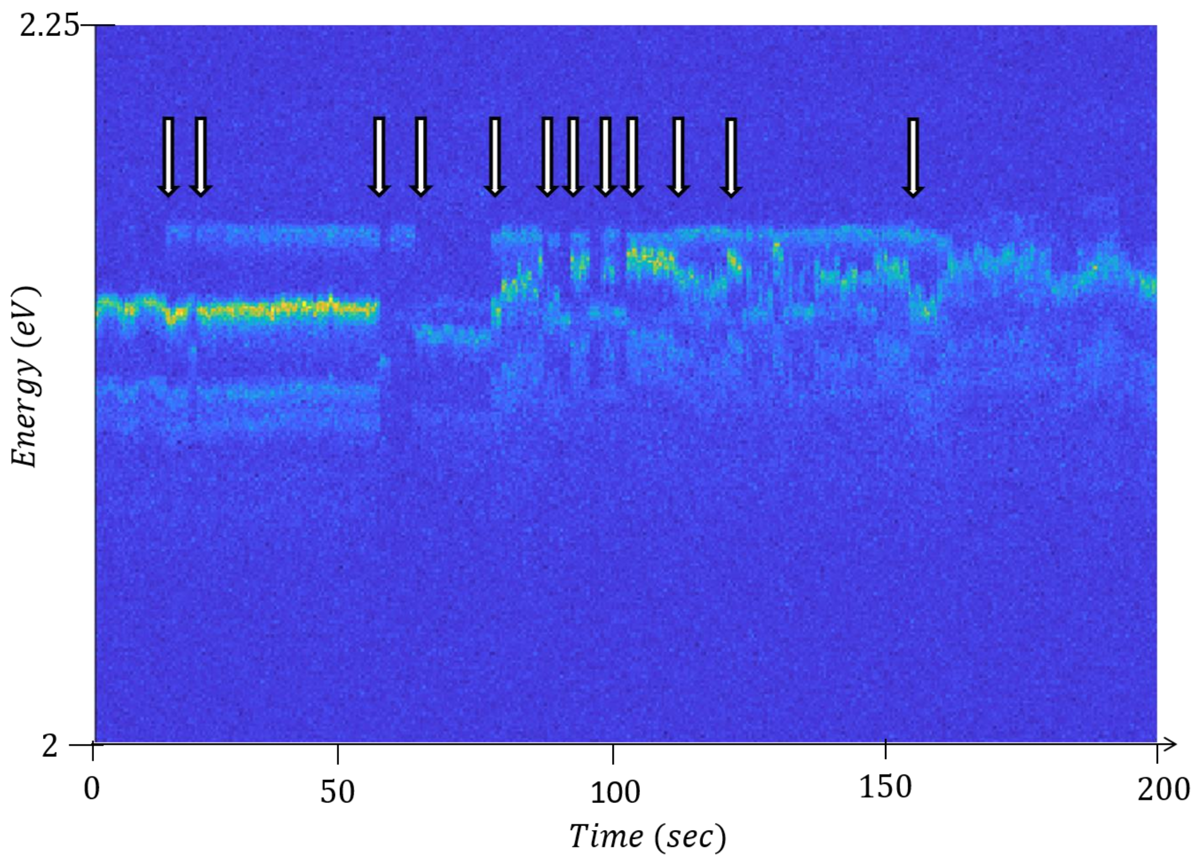

Figure S2. Simultaneous spectral shifts of the two QD comprising the CQDM. Example 2. The arrows mark the instances when simultaneous spectral shifts are seen.

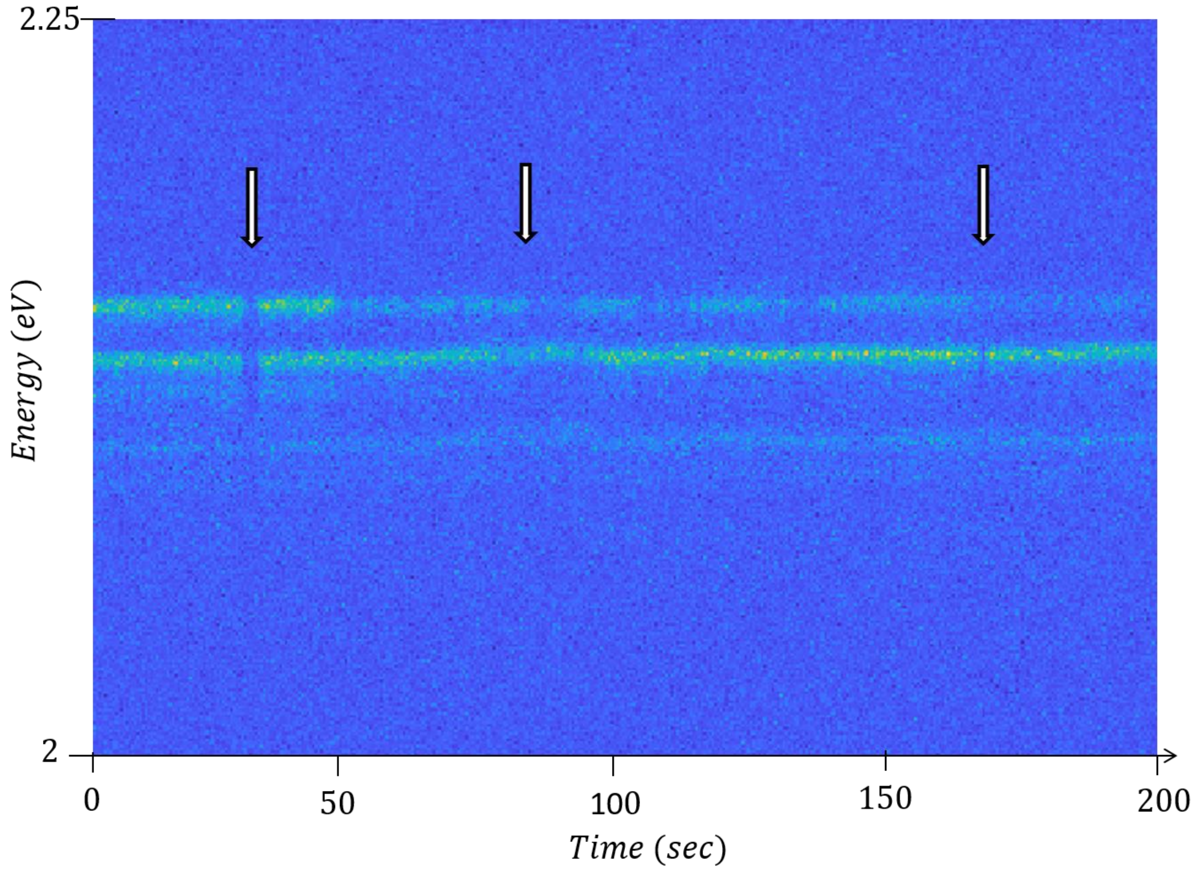

Figure S3. Simultaneous spectral shifts of the two QD comprising the CQDM. Example 3. The arrows mark the instances when simultaneous spectral shifts are seen.

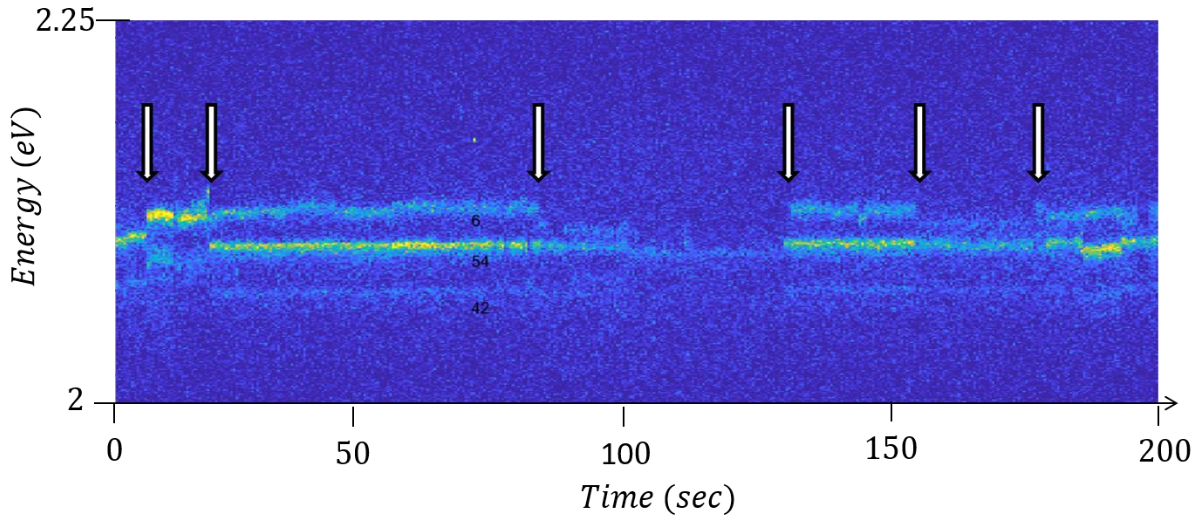

Figure S4. Simultaneous spectral shifts of the two QD comprising the CQDM. Example 4. The arrows mark the instances when simultaneous spectral shifts are seen. The numbers 6, 54 and 42 represent the polarization angle of the different lines.

Deducing the core and shell sizes of the CQDM analyzed in figure 4

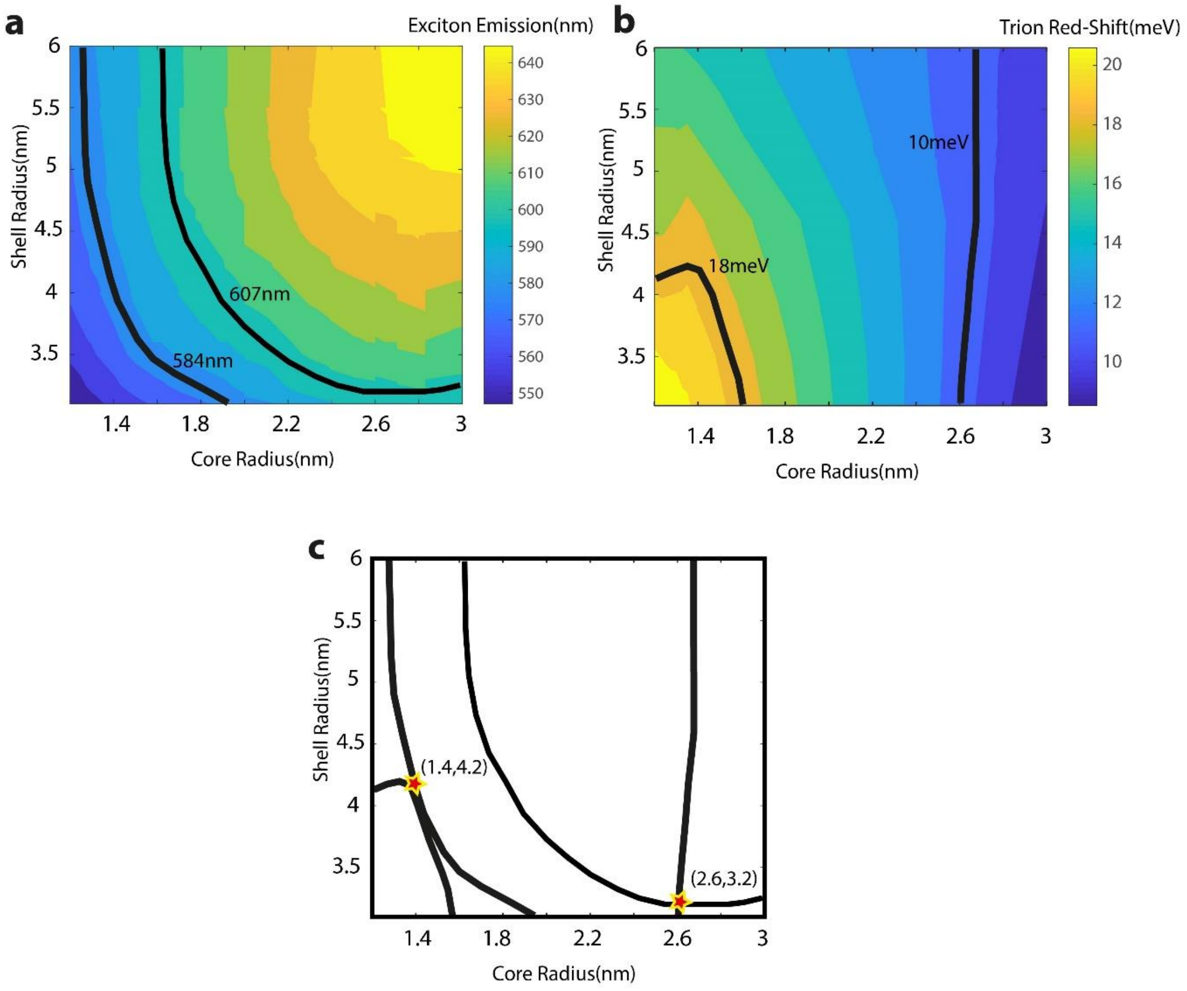

Figure S5. Deducing the core and shell sizes of the CQDM from figure 4. (a) Calculated emission position as a function of the core and shell radius. The experimental emission spectra of the two QDs comprising the CQDM are marked by the black lines. (b) Calculated negative trion energy red-shift as a function of the core and shell radius. The experimental negative trion energy shift of the two QDs comprising the CQDM are marked by the black lines. (c) The intersection of the lines for each of the QDs determines its dimensions.

Algorithm used to calculate the exciton, trion and tetron emission energies.

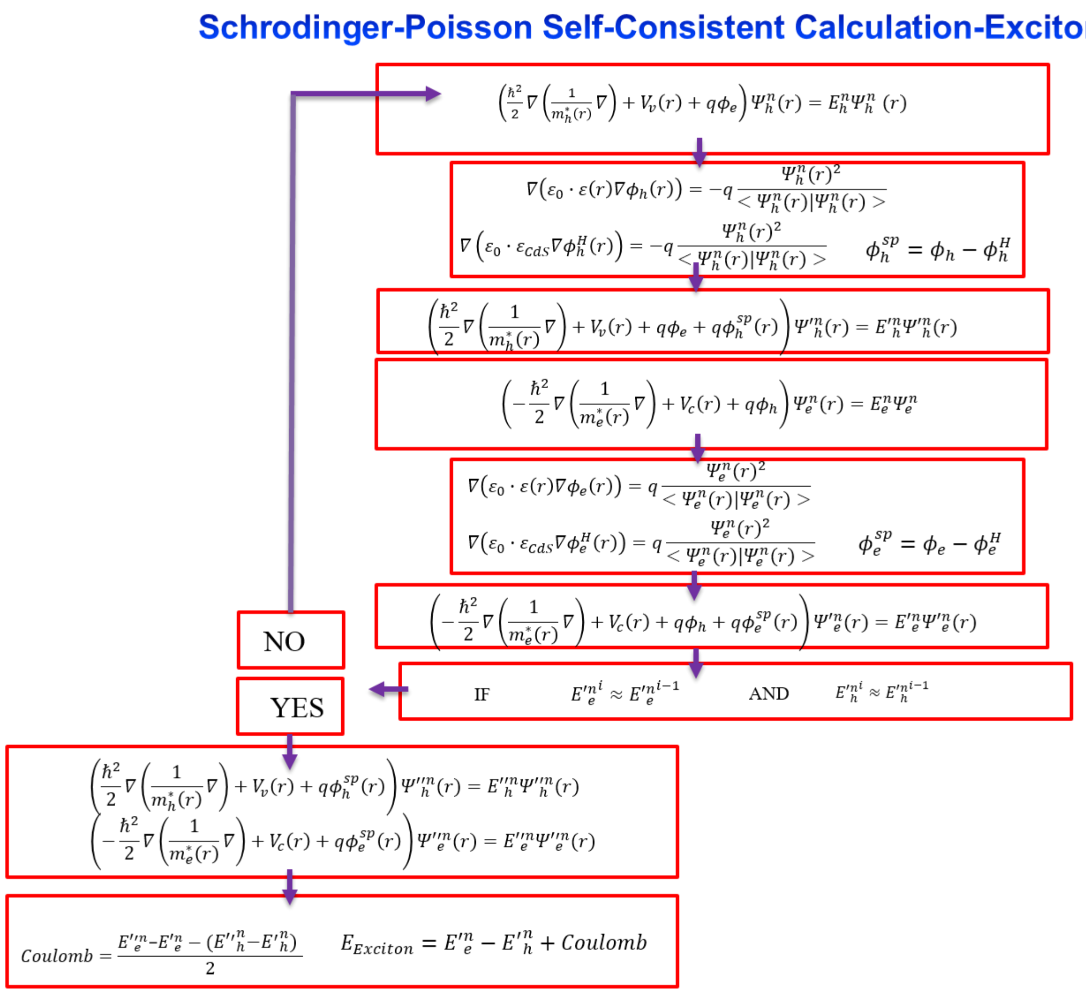

Figure S6. Algorithm used to calculate the exciton energy. $\Psi^n_{e/h}$ and $E^n_{e/h}$ represent the electron/hole wave-functions and energies in a potential induced by the band-offsets and the other charge carrier without taking into account the self-potential. $\Psi'^n_{e/h}$ and $E'^n_{e/h}$represent the electron/hole wave-function and energy in a potential induced by the band-offsets and the other charge carrier and taking into account the self-potential. $\Psi''^n_{e/h}$ and $E''^n_{e/h}$represent the electron/hole wave-function and energy in a potential made by the band-offsets and taking into account self-potential, using the last wave-function from the iteration, without the potential of the other charge carrier. This is calculated to discriminate between the potential from the other charge carrier to the self-potential. Only the potential from the other charge carrier shouldn't be taken twice. The entire computational space extends further from the QD boundary allowing for electron and hole wavefunctions to extend outside of the QD boundaries and decay into free space. We use von Neumann boundary-condition at the inner

(between core-shell) and outer boundaries of the QD in order to impose the Ben-Daniel-Duke condition. At the edge of the computational domain (around 30 nm away from the QD) we set the Dirichlet boundary-condition by setting the wavefunction to zero. Note that the self-potential was calculated in the same way as in ref.[1]. However, more sophisticated ways to calculate the self-potential can be found in ref.[2,3]. However, since these methods need more computational effort, we decided to use the simpler approximation above.

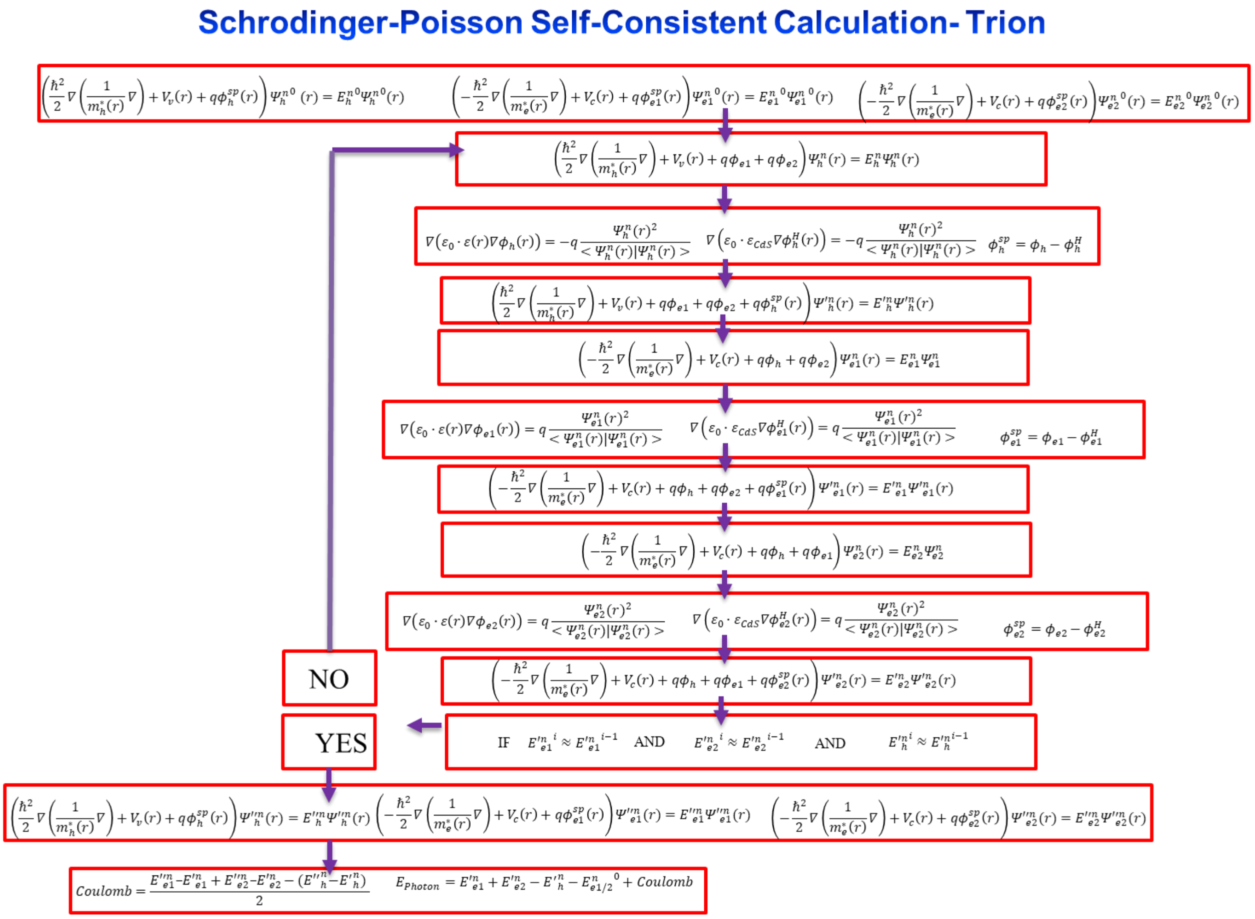

Figure S7. Algorithm used to calculate the trion energy. $\Psi_{e/h}^{n\ 0}$ and $\mathrm{E}_{e/h}^{n\ 0}$ represent the electron/hole wave-functions and energies in a potential induced by the band-offsets without the other charge carrier and with taking into account the self-potential. This is calculated in order to know what is the energy of the leftover electron after a recombination of an electron-hole pair in a trion state. $\Psi_{e/h}^{n}$ and $\mathrm{E}_{e/h}^{n}$ represent the electron/hole wave-function and the energy, respectively, in a potential induced by the band-offsets and the other charge carrier without taking into account the self-potential. $\Psi'^{n}_{e/h}$ and $\mathrm{E}'^{n}_{e/h}$ represent the electron/hole wave-function and its energy, respectively, in a potential induced by the band-offsets and the

other charge carrier and taking into account self-potential. $\Psi''^{n}_{e/h}$ and $E''^{n}_{e/h}$ represent the electron/hole wave-function and its energy, respectively, in a potential induced by the band-offsets and taking into account the self-potential, using the last wave-function from the iteration, without the potential of the other charge carrier. This is calculated to discriminate between the potential from the other charge carrier and the self-potential. Only the potential from the other charge carrier shouldn't be taken twice. Please note that both trions and tetrons are modeled here through a self-consistent method, which assumes that the wave function is the direct product of single-particle electron and hole functions as it is being modeled here.[4] However, Electrons are fermions. As such, they should be antisymmetric with respect to the permutation as represented using Slater determinants. Nevertheless, it will require much more computational efforts, beyond the scope of the present work.

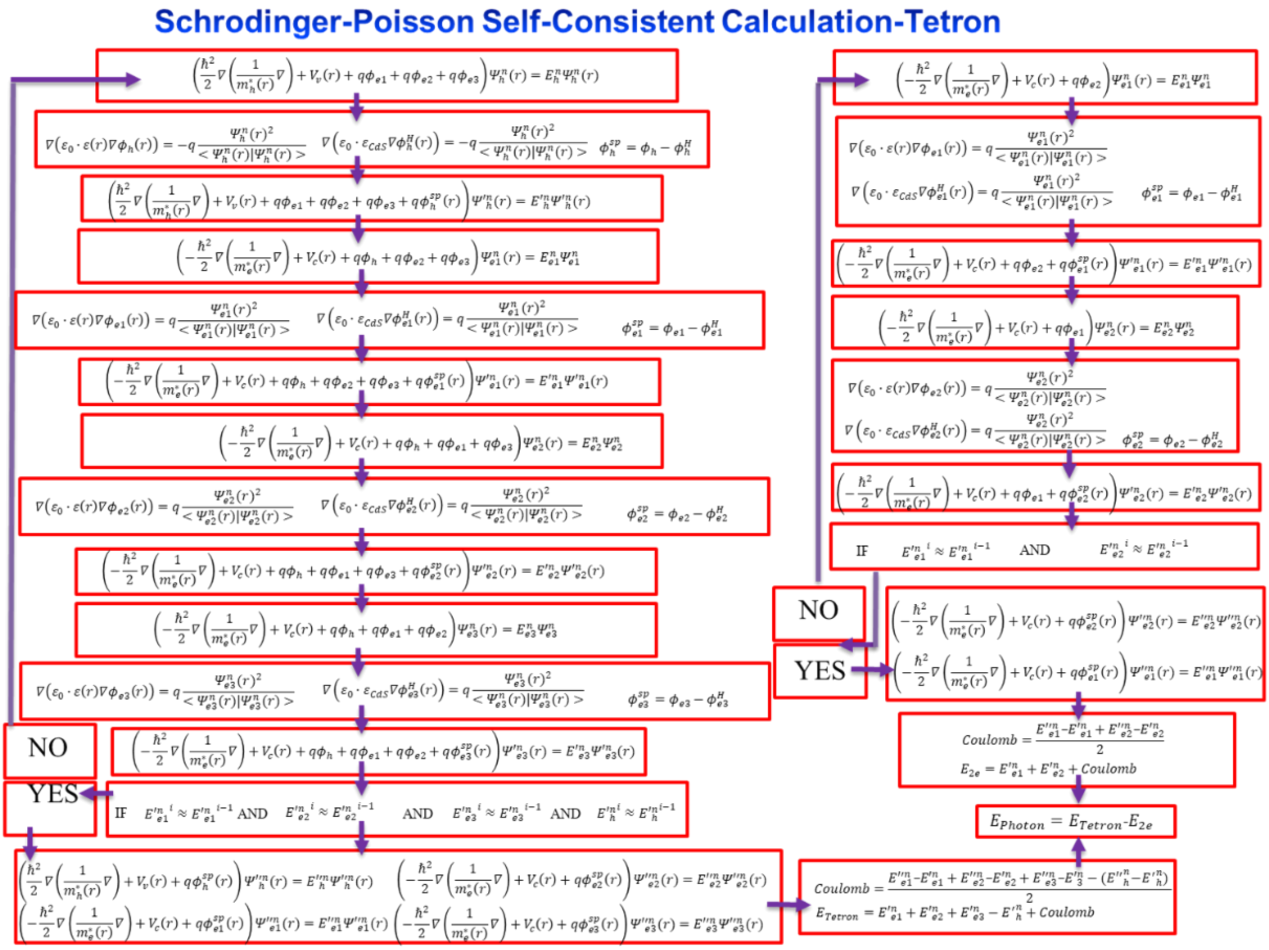

Figure S8. Algorithm used to calculate the tetron energy. $\Psi^{n}_{e/h}$ and $\mathrm{E}^{n}_{e/h}$ represent the electron/hole wave-function and energy, respectively, in a potential induced by the band-offsets and the other charge carrier without taking into account the self-potential. $\Psi'^{n}_{e/h}$ and $\mathrm{E}'^{n}_{e/h}$ represent the electron/hole wave-function and energy, respectively, in a potential induced by the band-offsets and the other charge carrier and taking into account the self-potential. $\Psi''^{n}_{e/h}$ and $\mathrm{E}''^{n}_{e/h}$represent the electron/hole wave-function and energy, respectively, in a potential induced by the band-offsets and taking into account the self-potential, using the last wave-function from the iteration, without the potential of the other charge carrier. This is calculated to discriminate between the potential from the other charge carrier and the self-potential. Only the potential from the other charge carrier shouldn't be taken twice. This second iteration is calculated in order to know what is the energy of the leftover two electrons, after a recombination of an electron-hole pair in the tetron state.

All cases where we see large spectra shifts in the simulations, even when the surface charge is on the other QD (e.g. fig. 5b at 8.4nm), are hybridization effects.

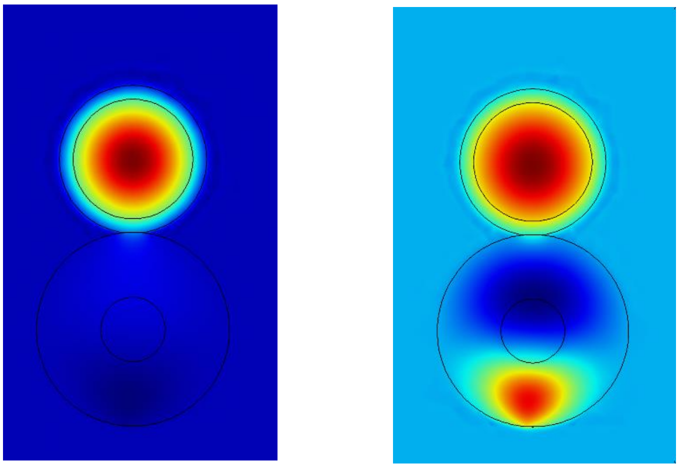

Figure S9. Hybridization effects in trion at QD2 with positive surface charge at 8.4nm. Plot of Ψ^{n}_{e} when the leftover electron is alone is presented on the right. In this case, the self-potential is low. However, after interaction with the other charge carriers it becomes localized (left figure). As a result the self-potential is higher.

Table I. Material parameters used in the simulations.

	CdSe	CdS	Environment	Units	Ref.
$\boldsymbol{V_c}$	1.76	1.86	5	[eV]	[4–8]
$\boldsymbol{V_v}$	0	-0.64	-5	[eV]	[4–8]
$\boldsymbol{m_e^*}$	0.112	0.21	1	m_0	[9]
$\boldsymbol{m_{h\perp}^*}$	0.48	0.376	1	m_0	[9]
$\boldsymbol{m_{h\parallel}^*}$	1.19	0.746	1	m_0	[9]
$\boldsymbol{\varepsilon_\perp}$	9.29	8.28	1	-	[9]
$\boldsymbol{\varepsilon_\parallel}$	10.16	8.73	1	-	[9]

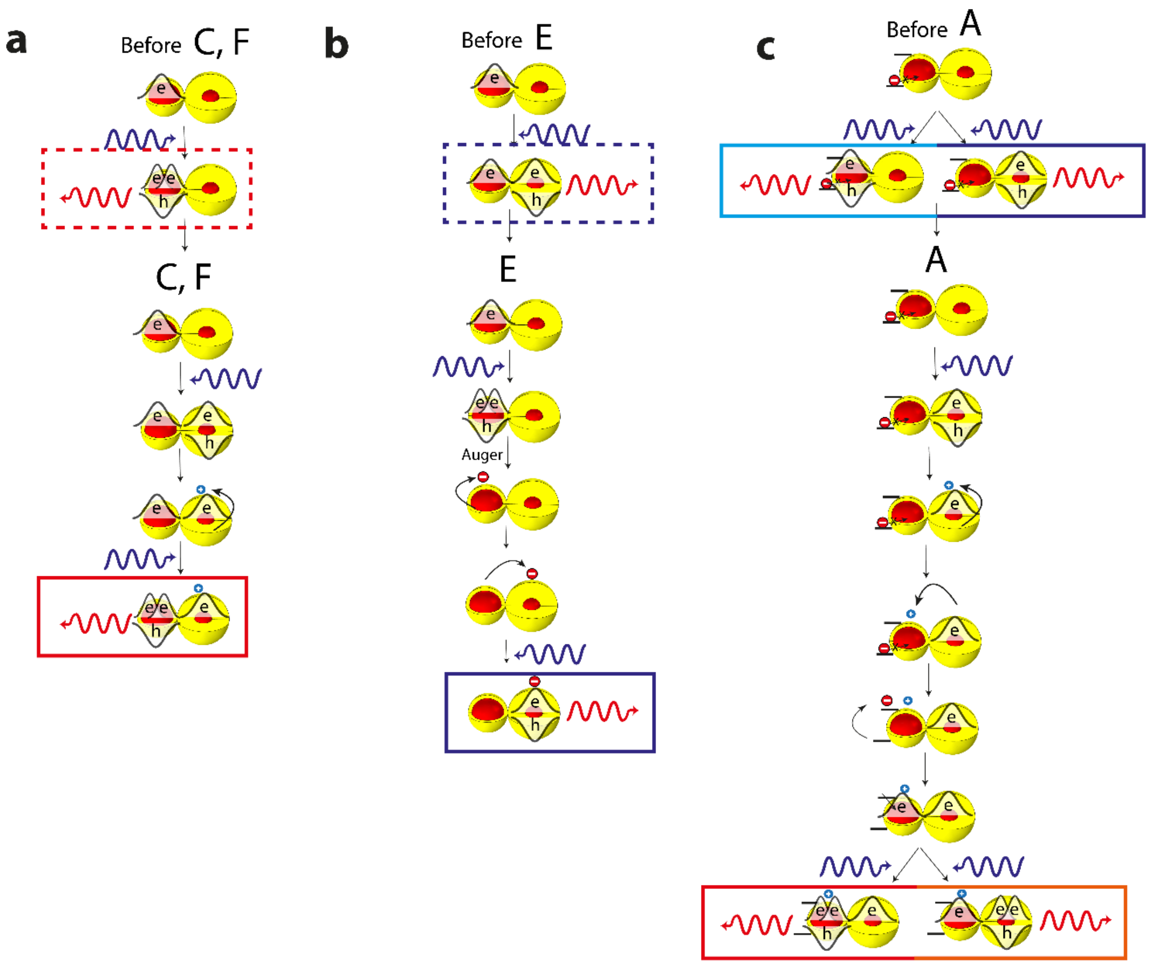

Figure S10. Assignment of the simultaneous spectral movements in the CQDM to surface charges. (a) in cases C and F (b) in case E and in case A (c). Colored rectangles highlighting the emission states before (dashed lines) and after (solid lines) the simultaneous spectral movement, respectively, and are color coded as the states in figure 5 in the main manuscript.

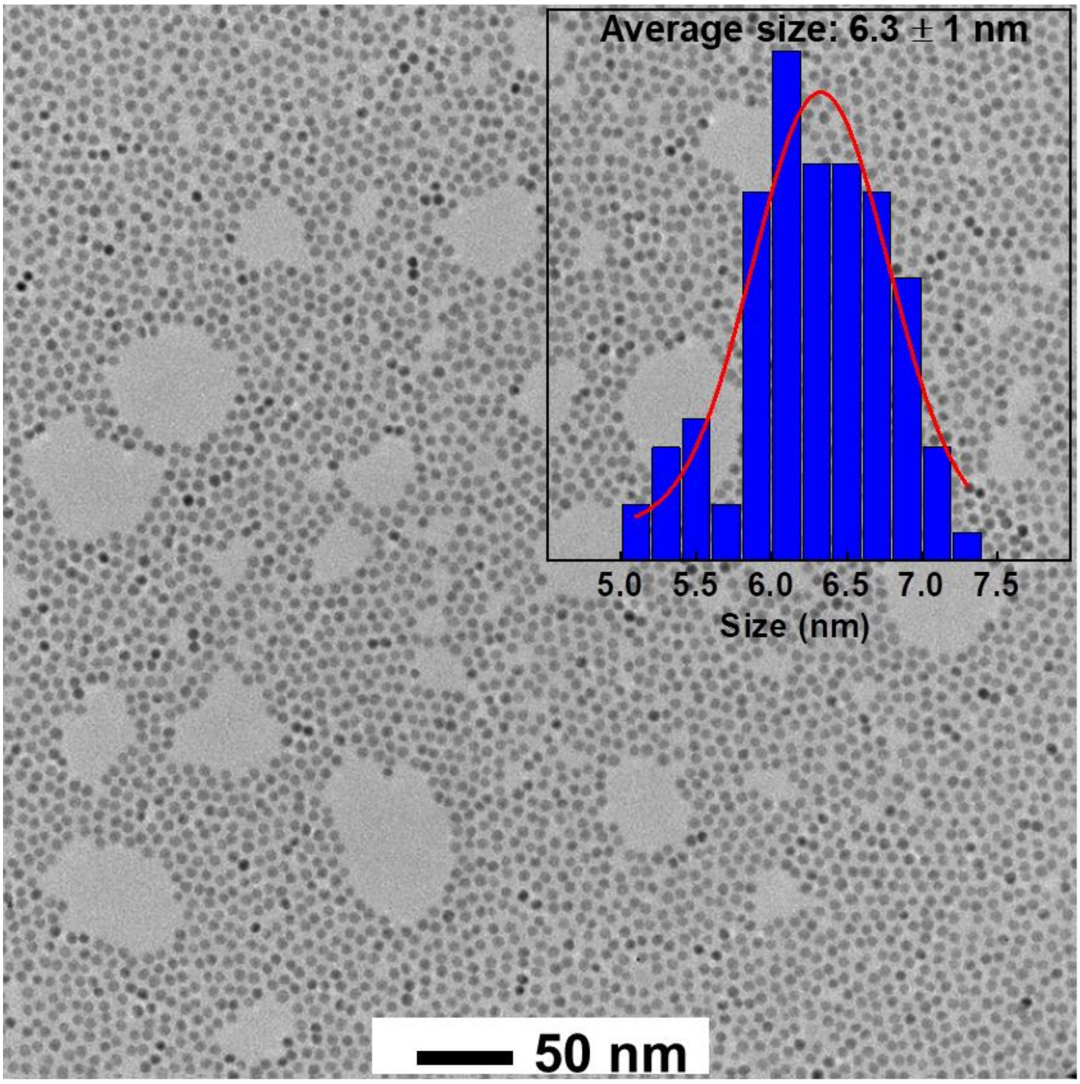

Figure S11. Monomers size dispersion analysis. The average diameter size of the monomers is $6.3 \pm 1\ nm$ (the error is because of the uncertainty to determine the boundaries of the nanocrystal in gray scale image). Around the average size the distribution is $\Delta = \pm 1.3 nm$ (ranging between 5-7.5 nm). Overall, the diameter of the monomers is $6.3 \pm 2.3\ nm$. The diameter of the two monomer comprising the dimer in figure 4, as analyzed in figure S5, is 6.4nm and 8.4nm inside the error limit.

References

1. Park, K., Deutsch, Z., Li, J. J., Oron, D. & Weiss, S. Single molecule quantum-confined Stark

effect measurements of semiconductor nanoparticles at room temperature. *ACS Nano* **6,** 10013–10023 (2012).

2. Movilla, J. L., Pi, M. & Planelles, J. Dielectric confinement in quantum dots of arbitrary shape within the local spin density approximation: Diluted regimes in elongated quantum dots. *J. Appl. Phys.* **108,** 64311 (2010).

3. Boda, D., Gillespie, D., Nonner, W., Henderson, D. & Eisenberg, B. Computing induced charges in inhomogeneous dielectric media: Application in a Monte Carlo simulation of complex ionic systems. *Phys. Rev. E - Stat. Physics, Plasmas, Fluids, Relat. Interdiscip. Top.* **69,** 10 (2004).

4. Grivas, C. *et al.* Single-mode tunable laser emission in the single-exciton regime from colloidal nanocrystals. *Nat. Commun.* **4,** (2013).

5. Steiner, D. *et al.* Determination of band offsets in heterostructured colloidal nanorods using scanning tunneling spectroscopy. *Nano Lett.* **8,** 2954–2958 (2008).

6. Sitt, A., Della Sala, F., Menagen, G. & Banin, U. Multiexciton engineering in seeded core/shell nanorods: Transfer from type-I to quasi-type-ll regimes. *Nano Lett.* **9,** 3470–3476 (2009).

7. Rainò, G. *et al.* Probing the wave function delocalization in CdSe/CdS dot-in-rod nanocrystals by time-and temperature-resolved spectroscopy. *ACS Nano* **5,** 4031–4036 (2011).

8. Muller, J. *et al.* Wave function engineering in elongated semiconductor nanocrystals with heterogeneous carrier confinement. *Nano Lett.* **5,** 2043–2049 (2005).

9. Christodoulou, S. *et al.* Band structure engineering via piezoelectric fields in strained anisotropic CdSe/CdS nanocrystals. *Nat. Commun.* **6,** 1–8 (2015).
